# Management of subdural effusion and hydrocephalus following decompressive craniectomy for posttraumatic cerebral infarction in a patient with traumatic brain injury: a case report

**DOI:** 10.1186/s12893-019-0489-5

**Published:** 2019-02-27

**Authors:** Ruhong Wu, Yun Ye, Tao Ma, Geng Jia, Huaping Qin

**Affiliations:** Department of Neurosurgery, The Third Affiliated Hospital of Soochow University, No.185, Juqian Road, Changzhou City, 213003 China

**Keywords:** Decompressive craniectomy, Hydrocephalus, Posttraumatic cerebral infarction, Subdural effusion, Subdural effusion with hydrocephalus

## Abstract

**Background:**

Subdural effusion with hydrocephalus (SDEH) is a rare complication of traumatic brain injury, especially following decompressive craniectomy (DC) for posttraumatic cerebral infarction. The diagnosis and treatment are still difficult and controversial for neurosurgeons.

**Case presentation:**

A 45-year-old man developed traumatic cerebral infarction after traumatic brain injury and underwent DC because of the mass effect of cerebral infarction. Unfortunately, the complications of traumatic subdural effusion (SDE) and hydrocephalus occurred in succession following DC. Burr-hole drainage and subdural peritoneal shunt were performed in sequence because of the mass effect of SDE, which only temporarily improved the symptoms of the patient. Cranioplasty and ventriculoperitoneal shunt were performed ultimately, after which SDE disappeared completely. However, the patient remains severely disabled, with a Glasgow Outcome Scale of 3.

**Conclusions:**

It is important for neurosurgeons to consider the presence of accompanying hydrocephalus when treating patients with SDE. Once the diagnosis of SDEH is established and the SDE has no mass effect, timely ventriculoperitoneal shunt may be needed to avoid multiple surgical procedures, which is a safe and effective surgical method to treat SDEH.

## Background

Posttraumatic cerebral infarction (PTCI) is a rare but well-known complication of traumatic brain injury (TBI), with an incidence ranging from 1.9 to 10.4% [1–5]. PTCI usually results in high mortality and is as an indicator of poor clinical outcome [5–7]. In patients with large infarctions and refractory elevated intracranial pressure (ICP), decompressive craniectomy (DC) is frequently performed as soon as possible to reduce ICP, decrease compression of cerebral vessels in the cerebral infarction, improve brain oxygen supply, improve outcomes and reduce mortality [8]. Although DC is a technically simple procedure, it is not without significant surgical complications [9–13]. Complications following DC include herniation of the cortex through the bone defect, subdural effusion (SDE), seizures, and hydrocephalus [14]. SDE is a relatively common complication following TBI [15]. Subdural effusion with hydrocephalus (SDEH) is a special case of SDE that is rarely reported as a complication of DC for TBI. Diagnosis and treatment remain difficult and controversial for neurosurgeons. Here, we report a TBI patient with SDEH following DC for PTCI. Although SDE disappeared after multiple unsuccessful surgical procedures, the patient remains severely disabled. The management was complex and difficult. Our goal is to present our experience in the management of SDEH, which may improve patient outcomes in the future.

## Case presentation

**Fig. 1:**
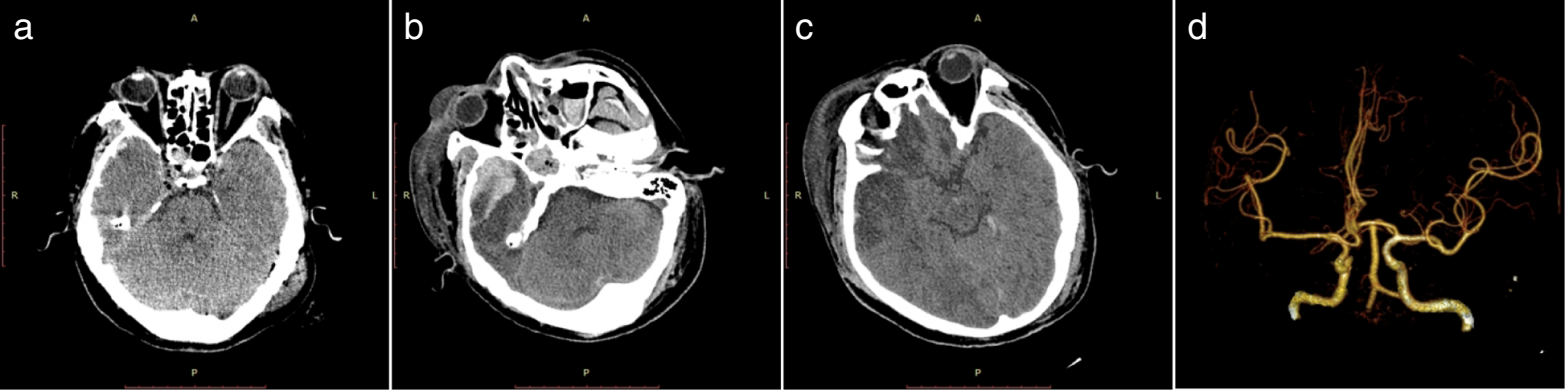
Posttraumatic brain computed tomography (CT) scan. **(a)** Brain CT scan on admission. **(b)** Brain CT scan on the second day after admission revealed a larger traumatic intracerebral hemorrhage. **(c)** Brain CT scan on the second day after admission revealed posttraumatic cerebral infarction. **(d)** Brain CT angiography

**Fig. 2:**
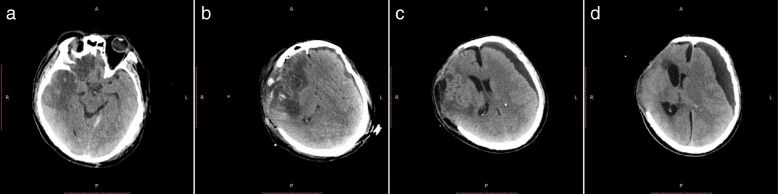
Brain CT scan before and after decompressive craniectomy. **(a)** Brain CT scan on the fourth day after admission. **(b)** Brain CT scan on the first day after decompressive craniectomy. **(c)** Brain CT scan two weeks after decompressive craniectomy. **(d)** Brain CT scan six weeks after decompressive craniectomy

**Fig. 3:**
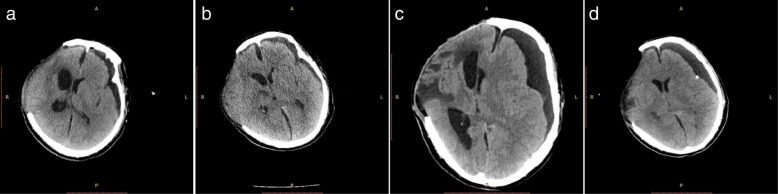
Brain CT scan after burr-hole drainage and subdural peritoneal shunt. **(a)** Brain CT scan on the first day after burr-hole drainage. **(b)** Brain CT scan after removal of the drainage tube. **(c)** Brain CT scan before subdural peritoneal shunt. **(d)** Brain CT scan after subdural peritoneal shunt

**Fig. 4:**
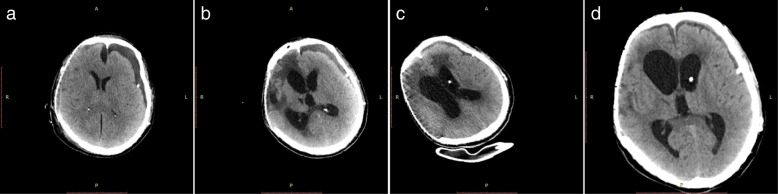
Brain CT scan after cranioplasty and ventriculoperitoneal shunt. **(a)** Brain CT scan on the first day after cranioplasty. **(b)** Follow-up brain CT scan after cranioplasty revealed incomplete resolution of the subdural effusion and re-dilation of the ventricles. **(c)** Brain CT scan on the first day after ventriculoperitoneal shunt. **(d)** Follow-up brain CT scan three months after ventriculoperitoneal shunt

A 45-year-old Chinese man involved in a road traffic accident was admitted to the emergency department presenting with a Glasgow Coma Scale (GCS) of 8. A computed tomography (CT) scan of his brain revealed a small, acute subdural hematoma in the right frontotemporal region and traumatic intracerebral hemorrhage in the right frontotemporal lobe with no mass effect (Fig. 1a). He initially received conservative treatment. The patient improved with a GCS of 12 on the second day after admission, and a follow-up brain CT scan revealed a larger traumatic intracerebral hemorrhage in the right temporal lobe (Fig. 1b) and a PTCI in the right frontotemporal lobe around the traumatic intracerebral hemorrhage (Fig. 1c). A brain CT angiography was subsequently performed, which revealed no abnormalities of the main intracranial arteries (Fig. 1d). Follow-up brain CT scans performed on the third and fourth day after admission revealed the gradually broadening scope of the PTCI (Fig. 2a). The PTCI showed a significant mass effect on the follow-up brain CT scan on the fourth day after admission, and the patient deteriorated again, with a GCS of 9, indicating the need for operation. He was transferred to the operating room and underwent a right DC. The patient remained intubated on postoperative day 1, and the postoperative follow-up CT scan showed the operation was successful, but a small amount of left SDE was revealed (Fig. 2b). Although we bandaged his head after the peak time of cerebral swelling, the left SDE enlarged progressively. Meanwhile, right subcutaneous effusion, interhemispheric SDE and ventricular dilation were detected on a follow-up CT scan 2 weeks after the DC (Fig. 2c). The patient began to deteriorate 6 weeks after DC, with a fixed left pupil, and a new brain CT scan revealed enlargement of the left SDE with a significant mass effect (Fig. 2d). He was transferred to the operating room immediately and underwent a left burr-hole drainage. The follow-up brain CT scan revealed the left SDE was reduced significantly (Fig. 3a), and the patient improved compared to his preoperative condition. A brain CT scan was taken after removal of the drainage tube (Fig. 3b). Unfortunately, the patient deteriorated again, with left eye mydriasis on the fifth day after drainage tube removal. An emergency brain CT scan detected a significant mass effect from SDE again (Fig. 3c), and he was transferred to the operating room and underwent a left subdural peritoneal shunt (SPS). Although the ventricle narrowed, the SDE did not disappear completely (Fig. 3d). He underwent a cranioplasty 20 days after the SPS (Fig. 4a), but the follow-up brain CT scan revealed that the SDE did not resolve completely and the ventricle was dilated again (Fig. 4b). Ultimately, we conducted a ventriculoperitoneal shunt (VPS) 75 days after the cranioplasty (Fig. 4c). During the VPS placement, we connected the ventricular shunt tube to the valve of the SPS with a Y-shaped connection tube. A follow-up brain CT scan three months after the VPS placement showed that the SDE disappeared but the ventricular dilation still remained (Fig. 4d). Ultimately, he remains severely disabled, obeying simple commands and with a Glasgow Outcome Scale of 3 when transferred to the rehabilitation hospital.

## Discussion and conclusion

PTCI is a rare but well-recognized complication of TBI, aggressive treatments such as DC should be considered in PTCI patients with large infarctions and refractory elevated ICP [16]. DC, which transforms the closed cranial cavity into an open system that provides additional compensatory space for swollen brain tissue, has been widely used as a treatment for refractory elevated ICP [17, 18]. SDE and hydrocephalus are not rare and can occur secondary to DC. The incidence of SDE is 21.3% after DC for TBI [13]. The incidence of post-traumatic hydrocephalus in patients with DC for TBI is 10 to 40% [10, 12, 13, 19].

SDE is defined as cerebrospinal fluid (CSF) accumulation in the subdural space [20]. SDEH has been described after aneurysm rupture and subarachnoid hemorrhage, [21, 22] head injuries [22] and neurosurgery, [23–25] which is a special circumstance of SDE. The mechanisms of SDEH after DC might include the tearing of some part of the arachnoid membrane, particularly the basal cisterns or lamina terminalis, allowing CSF to flow into this compartment [26]. After DC, the ICP is imbalanced in each brain cavity; the formation of SDE requires less pressure than the enlargement of ventricles [24]. Furthermore, hydrocephalus is a common complication secondary to DC for TBI. SDEH will subsequently occur while the ventricles communicate with the subdural space and the CSF circulates inappropriately.

When treating patients with SDEH, neurosurgeons must differentiate SDEH from subdural hygromas. In SDEH, SDE can communicate freely with the subarachnoid space, while subdural hygromas cannot. When ventriculomegaly occurs before the formation of SDE, CT scans reveal dilatated lateral ventricles and periventricular lucency in most patients [23]. However, most SDE appears earlier than hydrocephalus, and as time goes on, the SDE can regress or exist simultaneously with hydrocephalus [20]. Hence, distinguish subdural hygroma and SDEH prior to ventriculomegaly is difficult. Previous work indicated that the CT value was significantly lower for SDEH than that for subdural hygroma at the same volume of fluid [27]. Enhanced CT and magnetic resonance imaging reveal that SDEH does not have an enhancement capsule, while subdural hygroma shows an enhancement capsule [28]. CT cisternography is another method to differentiate SDEH and can be used to detect the communication between the subdural space and the ventricles [23]. However, this approach has proven useless in complex cases of SDEH [26]. In regard to the diagnosis of hydrocephalus, some neurosurgeons suggest measuring ventricle size with a modified frontal horn index (mFHI), that is, the largest width of the frontal horns divided by the bilateral cortical distance in the same plane [21]. Patients with an mFHI greater than 0.33 are more likely to have SDEH rather than subdural hygroma, [21] indicating the potential value of the mFHI in diagnosing SDEH.

Some studies have proposed classifying and treating traumatic SDE based on its pathophysiology and the mass effect [20, 29]. Group Ia represents a simple SDE with no mass effect and with no hydrocephalus. Group Ib represents simple SDE with no mass effect and with hydrocephalus. Group IIa represents SDE with mass effect and with no hydrocephalus. Group IIb represents SDE with mass effect and with hydrocephalus. Group Ia and Ib do not require surgical intervention, while Group IIa and Group IIb require surgical intervention. Studies have indicated that burr-hole drainage or SPS can temporarily improve the symptoms of patients with symptomatic SDEH but with a high likelihood that subsequent VPS will be needed. The decision to treat an SDEH with a VPS when the SDE has an obvious mass effect is difficult for many neurosurgeons, who often prefer to wait until the SDE has no mass effect and the free flow between subdural space and enlarged ventricles is established, [23] especially in patients with a DC. Thus, patients may often receive repeated surgical treatments such as burr-hole drainage and SPS due to SDE, which misses the optimal surgical window and increases the risk of complications, including CSF leak and subsequent infection, and delay of VPS will aggravate neurologic function [26].

Certain measures can also decrease the incidence of SDEH. Some neurosurgeons have suggested duraplasty as a means to avoid the disturbance to CSF circulation following DC, which may decrease the incidence of SDE [13, 30]. Bandaging the head to avoid brain herniation after the peak time of cerebral swelling is another measure to prevent SDE [31]. Some neurosurgeons have reported delayed cranioplasty associated with hydrocephalus and suggested that early cranioplasty may prevent the alteration in CSF hydrodynamics after DC to promote spontaneous improvement of hydrocephalus [13, 31–34].

SDEH as a rare complication after DC has been well described in the past few years. However, at present, there are different opinions regarding the best treatment strategy. An increasing number of neurosurgeons tend to adopt VPS, which can eliminate the SDE and ultimately improve symptoms of hydrocephalus. In this case, although the SDE was ultimately resolved, we may have missed the best time window during which to treat SDEH. In this patient, in addition to the complication of SDEH after DC due to PTCI, in the course of treating SDEH, we adopted almost all the current methods of treating SDEH, including conservative treatment (bandaging the head), burr-hole drainage, SPS, cranioplasty and VPS. This condition is rarely reported in previous literature. The purpose of this case description is not only to draw clinical lessons but also to enable neurosurgeons to better understand SDEH and to provide experience for future treatments.

SDEH is a rare complication of TBI, especially after a DC for PTCI. Accurate diagnosis of SDEH and differentiation from other subdural collections are crucial. Burr-hole drainage and SPS can only temporarily improve the symptoms of patients with SDEH, while a VPS might ultimately be necessary in patients with SDEH. VPS is a safe and effective surgical method to treat SDEH under the condition of the SDE with no mass effect. Once the diagnosis of SDEH is established, and the SDE has no mass effect, a VPS may need to be implanted quickly to avoid multiple surgical procedures. In the future, more cases and studies need to explore whether VPS or SPS plus VPS can be reasonably performed during the first surgery to avoid a second surgery when treating patients with SDEH, especially when the SDE has a mass effect.

## Abbreviations

CSF: Cerebrospinal fluid
CT: Computed tomography
DC: Decompressive craniectomy
GCS: Glasgow Coma Scale
ICP: Intracranial pressure
mFHI: modified frontal horn index
PTCI: Posttraumatic cerebral infarction
SDE: Subdural effusion
SDEH: Subdural effusion with hydrocephalus
SPS: Subdural peritoneal shunt
TBI: Traumatic brain injury
VPS: Ventriculoperitoneal shunt
